# Bats and zoonotic viruses: can we confidently link bats with emerging deadly viruses?

**DOI:** 10.1590/0074-02760150048

**Published:** 2015-02

**Authors:** Ricardo Moratelli, Charles H Calisher

**Affiliations:** 1 Fiocruz Mata Atlântica, Rio de Janeiro, RJ, Brasil, Fiocruz Mata Atlântica, Rio de Janeiro, RJ, Brasil; 2 Arthropod-borne and Infectious Diseases Laboratory, Department of Microbiology, Immunology and Pathology, College of Veterinary Medicine and Biomedical Sciences, Colorado State University, Fort Collins, CO, USA

**Keywords:** bat-borne viruses, biosafety, Chiroptera, bat immunology, emerging infectious diseases, zoonosis

## Abstract

An increasingly asked question is 'can we confidently link bats with emerging viruses?'. No, or not yet, is the qualified answer based on the evidence available. Although more than 200 viruses - some of them deadly zoonotic viruses - have been isolated from or otherwise detected in bats, the supposed connections between bats, bat viruses and human diseases have been raised more on speculation than on evidence supporting their direct or indirect roles in the epidemiology of diseases (except for rabies). However, we are convinced that the evidence points in that direction and that at some point it will be proved that bats are competent hosts for at least a few zoonotic viruses. In this review, we cover aspects of bat biology, ecology and evolution that might be relevant in medical investigations and we provide a historical synthesis of some disease outbreaks causally linked to bats. We provide evolutionary-based hypotheses to tentatively explain the viral transmission route through mammalian intermediate hosts and to explain the geographic concentration of most outbreaks, but both are no more than speculations that still require formal assessment.

Taxonomically, bats are grouped in the order Chiroptera (Gr. *cheir*, hand; *pteron*, wing) and, as the name suggests, they have morphological and physiological adaptations for powered flight. These characteristics and the ecosystem services they provide set them apart from all other mammals. Other aspects of their natural history, ecology, biology and evolution also distinguish them from other mammals, but none has been more emphasised recently than their potential link with human diseases, particularly those caused by zoonotic viruses (Wibbelt et al. 2010, Kupferschmidt 2013, Luis et al. 2013, Plowright et al. 2015).

Although many bat viruses have been associated with human, livestock and wild animal diseases, the sudden appearance of newly recognised viruses causing dreadful diseases has been surprising, sometimes shocking to the scientific and medical communities. Humans have responded to these diseases in rapid and often unprepared and disorganised ways. In part, this has occurred because bat biologists have been in denial regarding their favourite mammals due to the fear of further damage to the illogical and false reputation of bats as vampires and as carriers of rabies virus. This has been a disservice to both our understanding of the biology of bats and to the various medical and research communities and their patrons. False images of bats engendered by entertaining, but preposterous motion pictures, alarmist stories in the popular press and ancient superstitions have been embedded in the minds of the public and have impeded education and acceptance of these remarkable, useful and widely diverse animals. Thus, until relatively recently, studies of the relationships of bats and viruses have lagged far behind studies of viruses in humans, livestock, birds, other wild vertebrates and arthropods (Calisher et al. 2006).

With human encroachment on natural areas intensifying, particularly in those with high biological richness (Myers et al. 2000, Ceballos & Ehrlich 2006) and bats recognised as important reservoirs of zoonotic viruses (Luis et al. 2013), considerable concern and interest have been expended on them. In addition, bats of some species adapt well to disturbed habitats (Jones et al. 2009), so that these vertebrates have quickly become a focus of epidemiologic studies. However, most epidemiologists know bats only from outdated information or know very little or nothing about their biology, ecology and evolution. A similar situation occurs regarding bat biologists, many of whom know little or nothing about diseases ostensibly associated with bats. In an attempt to fill the gaps in knowledge about bats in general, bats as virus hosts and bat diseases, we review many aspects of bat biology, ecology and evolution, which we expect will enlighten medical investigations. In addition, we provide a historical synthesis of disease outbreaks causally linked to bats and which have caused isolated deaths or severe outbreaks of diseases. By linking these aspects, we then attempt to fit together some pieces of this puzzle. We anticipate that this review will stimulate collaborations between bat biologists and medical researchers in field and laboratory investigations concerning both basic and applied research.

## Biology, diversity and evolution of bats

*Diversity, distribution and biology* - Bats vary widely in size and form. Their body masses range from 2 g in the bumblebee bat [*Craseonycteris thonglongyai* (Craseonycteridae), the second smallest mammal known] to 1 kg in some flying-foxes [*Pteropus* spp (Pteropodidae)], whose wingspans can reach 2 m (Wilson 1997). Among mammals, bats are second only to rodents in species richness, with more than 1,300 species recognised currently (Fenton & Simmons 2015). This total comprises almost one fifth of the world's mammal species, with more than 175 genera (Simmons 2005, Wilson & Reeder 2005) arranged in 20 families.

Bats are distributed widely in the world, occurring on all continents, except Antarctica. They are the second most widespread order of mammals, surpassed only by Primates due to the wide distribution of humans. Due to their ability to fly, they have colonised many oceanic islands and on some they are the only native mammals (Koopman 1994). Among the families recognised currently (Fenton & Simmons 2015), Emballonuridae, Molossidae and Vespertilionidae occur in both the New and Old World, Cistugidae, Craseonycteridae, Hipposi- deridae, Megadermatidae, Miniopteridae, Mystacinidae, Myzopodidae, Nycteridae, Pteropodidae, Rhinolophidae and Rhinopomatidae occur only in the Old World, and Furipteridae, Mormoopidae, Natalidae, Noctilionidae, Phyllostomidae and Thyropteridae occur only in the New World. Table I provides information on distribution and diet of bats by family. The evolution of flight - the most peculiar characteristic of bats and one of the most important for their wide distribution - may have had effects on some aspects of the evolution of the immune system and the metabolism of bats, allowing them to host so many viruses (O'Shea et al. 2014, Brook & Dobson 2015).

**TABLE I:** Synthesis of the distribution and diet of bats by family

Family (number of species)^a^	Common names^b^	Distribution	Feeding items
Cistugidae (2)	Winged-gland bats	Southern Africa	Insects
Craseonycteridae (1)	Bumblebee bats	Thailand, Burma	Insects, spiders
Emballonuridae (54)	Sheath-tailed bats	Pantropical	Insects, occasionally fruits
Furipteridae (2)	Smoky bats	Neotropics	Insects
Hipposideridae (9)	Old World leaf-nosed bats	Old World tropics and subtropics	Insects
Megadermatidae (5)	False vampire bats	Old World tropics	Arthropods, small vertebrates
Miniopteridae (29)	Bent-winged bats	Old World tropics and subtropics	Insects
Molossidae (113)	Free-tailed bats	Pantropical	Insects
Mormoopidae (10)	Moustached bats	Neotropics	Insects
Mystacinidae (2)	New Zealand short-tailed bats	New Zealand	Insects and other arthropods; also feeding on nectar and fruits
Myzopodidae (2)	Old World disk-winged bats	Madagascar	Insects
Natalidae (12)	Funnel-eared bats	Neotropics	Insects
Noctilionidae (2)	Bulldog bats	Neotropics	Insects; 1 specie feeds on fishes
Nycteridae (16)	Slit-faced bats	Old World tropics	Insects, spiders, scorpions; 1 specie feeds on small vertebrates
Phyllostomidae (204)	New World leaf-nosed bats	Neotropics	Animals and plants
Pteropodidae (198)	Old World fruit bats	Old World tropics and subtropics	Fruits, nectar, pollen
Rhinolophidae (97)	Horseshoe bats	Old World tropics and subtropics	Insects
Rhinopomatidae (6)	Mouse-tailed bats	Old World tropics	Insects
Thyropteridae (5)	New World disk-winged bats	Neotropics	Insects
Vespertilionidae (455)	Vesper bats	Cosmopolitan	Most species feed exclusively on insects, but a few also feed on other arthropods (like scorpions), fishes and small birds

*a*,* b*: scientific and vernacular family group names and numbers of species follow Fenton and Simmons (2015).

Bats are nocturnal mammals (Rydell & Speakman 1995), with most of them spending the day in roosts and foraging from dusk to dawn. In general, they have one or two peaks of activity throughout the night (Fenton 1983). Tropical bats are active year-round and those that live in temperate zones either migrate or hibernate to avoid unfavourable environmental conditions (Fenton 1983, Wilson 1997).

**Fig. 1A:**
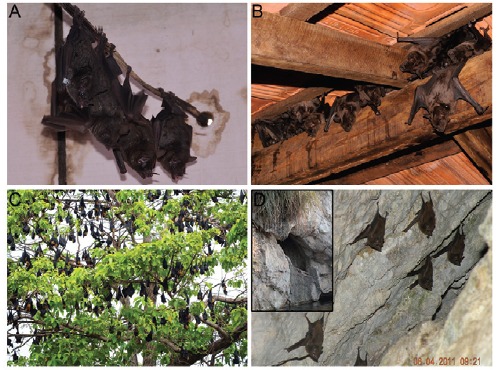
Seba's short-tailed bats [Carollia perspicillata (Phyllostomidae)]; B: greater spear-nosed bats [Phyllostomus hastatus (Phyllostomidae)] using human-made constructions as day roosts; C: flying-foxes (Pteropidae) hanging on trees during the day; D: lesser dog-like bats [Peropteryx macrotis (Emballonuridae)] roosting in the crevice of a rock in the edge of a river (the inset shows the entrance to the crevice). A and B are courtesy of A Pol (Federal Rural University of Rio de Janeiro, Brazil), C was acquired from Shutterstock Inc and D is courtesy of E Rubião (self-employed contractor). Photographers are the copyright holders of the images.

Bats exploit a great variety of roosts. They can use hollows (caves, mines, tree trunks, buildings etc.), crevices and foliage as day roosts (Fig. 1) and many frugivorous and insectivorous bats are known to use night roosts for eating and grooming. Partially eaten fruits and insects and droppings can be found on the ground of their night roosts (Fenton 1983) and they are good indicators of the items included in their diet. Fruits partially eaten by bats have been used to link bats with emerging zoonotic viruses (Chua et al. 2002).

Bat reproduction is similar to that of other mammals, but mating and duration of breeding season are strongly influenced by hibernation and migration. In hibernating bats of various species, copulation generally occurs in the fall, either with ovulation and fertilisation occurring immediately, followed by a slow development of the fetus throughout the winter and birth in the spring, or with sperm storage until spring, after which ovulation, fertilisation and foetal development occurs. In migratory bats, mating is generally concentrated in the early spring, after they reach their summer roosts. These bats and several others that do not need to migrate or hibernate, but that live in areas with food availability varying seasonally, reproduce once a year (monoestrus), whereas others facing less marked seasonal variation or having food resources available year-round generally reproduce twice or more a year (polyestrus). The former pattern is characteristic of most insectivorous bats from temperate regions, whereas the latter is common in most frugivorous bats living in tropical regions. Gestation averages about two months and bats of most species produce a single young per litter, but a few produce twins and a very few produce three or four young at a time (Wilson 1997). Halpin et al. (2000) isolated Hendra virus from uterine fluid and foetal tissues of bats and Drexler et al. (2011) found evidence of virus amplification during colony formation and after parturition.

Life span usually decreases with body size for mammals, with larger animals living longer, but bats are exceptions to this rule; longevity in bats was reviewed by Wilkinson and South (2002). Bats can live 3.5 times longer, on average, than non-volant placental mammals with similar body size (Wilkinson & South 2002) and, considering their size, they live longer than any other mammal (Bouliere 1958, Austad & Fischer 1991). The maximum age varies greatly from species to species, but while small rodents live in the wild about one-two years, a bat with similar body size can live more than 30 years. A Brandt's bat [*Myotis brandtii* (Vespertilionidae)] from Siberia was recaptured 41 years after the first capture (Podlutsky et al. 2005), but records of bats older than 30 years are known only for bats of five species in the wild (Wilkinson & South 2002). Genome and transcriptome analyses have revealed unique sequence changes that appear to contribute to both the small body size and the long lifespan of bats (Seim et al. 2013).

Species richness increases toward the tropics and in most tropical areas bat diversity is higher than that of any other group of mammals. As an example, in a 3-km radius of a rainforest in northern French Guiana, bats of at least 78 species co-exist in the area and the diversity analyses indicate that the fauna is not fully sampled (Simmons & Voss 1998). In the same area, 64 non-volant mammals have been recorded, including 22 rodents, 12 marsupials, 10 carnivores, nine xenarthrans, six primates and five ungulates (Voss et al. 2001). Bats also are the most abundant mammals in several tropical forests and the wild vertebrates that more often interact with humans, although most people do not realise it.

Bats of some species also form large aggregations and a few of them form the largest aggregations of mammals in the world. Old World fruit bats (Pteropodidae) of various species constitute colonies of hundreds of thousands to millions of bats that aggregate on exposed tree branches (Mickleburgh et al. 1992, Kunz & Pierson 1994). In Bracken Cave, central Texas, about 20 million Mexican free-tailed bats [*Tadarida brasiliensis mexicana* (Molossidae)] form the largest warm-blooded non-human vertebrate colony in the world.

**Fig. 2A-F:**
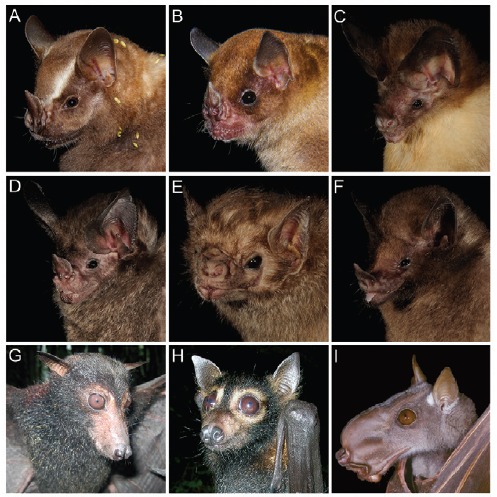
New World leaf-nosed bats of family Phyllostomidae, including frugivores - the great-eating fruit bat (Artibeus lituratus) (A) with seeds in the fur and the little yellow-shouldered bat (Sturnira lilium) (B), insect eaters - the common big-eared bat (Micronycteris cf. microtis) (C) and the stripe-headed round-eared bat (Tonatia saurophila) (D), a blood feeder - the white-winged vampire bat (Diaemus youngi) (E) and a nectar feeder - the Thomas's nectar bat (Hsunycteris thomasi) (F); G-I: Old World fruit bats of family Pteropodidae, including the black flying-fox (Pteropus alecto) (G), the spectacle flying-fox (Pteropus conspicillatus) (H) and the hammer-headed fruit bat (Hypsignathus monstrosus) (I). A and B are courtesy of RLM Novaes (Oswaldo Cruz Foundation, Brazil), C-F are courtesy of A Pol (Federal Rural University of Rio de Janeiro, Brazil), G and H are courtesy of A Breed (Animal and Plant Health Agency, Addlestone, Surrey, United Kingdom) and I is courtesy of Jakob Fahr (Max Planck Institute for Ornithology, Germany). Photographers are the copyright holders of the images

Although bats are outnumbered by rodents in species richness, they are first among mammals (and probably among vertebrates) in dietary diversity (Fenton & Simmons 2015) which includes remarkable adaptations to explore an array of different animal and plant food items (Wilson 1973, 1997, Altringham 1996, 2011). This variety of behaviours has been arranged into eight main feeding categories: fruit eaters, flower feeders, aerial insectivores (those that capture insects in flight), foliage gleaners (capture insects on the ground), carnivores (feed on small terrestrial vertebrates, including birds, frogs and mammals), fish eaters, blood feeders and omnivores (Wilson 1973). Phyllostomids - also called New World leaf-nosed bats and restricted to the Neotropics - include representatives classified in quite distinct categories (Fig. 2A-F), with insect foliage gleaners, carnivores, blood feeders, nectar feeders and fruit eaters; the latter mainly grouped into the subfamilies Stenodermatinae, Carolliinae and Rhinophyllinae. Pteropodids - the fruit bats restricted to the Old World tropics (Old World fruit bats) - also include fruit eaters and nectar feeders (Fig. 2G-I). Except for representatives of these two families, bats are primarily animal feeders, generally insect eaters. Some pteropodid and phyllostomid bats that feed on fruits reject the fibres and other indigestible components. They chew and ingest the pulp and juices and drop the fibrous material and larger seeds that can be found on the ground below their night roosts. They swallow smaller seeds that pass quickly and undamaged through the bat's gastrointestinal tract (ca. 20 min). Using a similar strategy to increase feeding efficiency, insect-eaters from various families may cull the insects they prey on, consuming the most nutritional part (the abdomen) and discarding wings, head and appendages (Fenton 1983, Kunz et al. 2011).

*Evolutionary history* - Chiroptera is a middle Paleocene or early Eocene lineage of placentals (Simmons et al. 2008, O'Leary et al. 2013). This means that bats have evolved apart from other mammalian lineages for more than 50 million years. Although they have a long history of isolation from other mammals, understanding their evolutionary relationships with other groups of mammals and even between bats of different families, can provide important clues in investigating physiological aspects that may favour disease spillover events.

Bats comprise a monophyletic lineage that has evolved within the superorder Laurasiatheria (Van Den Bussche & Hoofer 2004, O'Leary et al. 2013). This superorder also includes the order Lipotyphla [formerly Insectivora, but excluding tenrecs (Tenrecidae) and golden moles (Chrysochloridae)], Pholidota (pangolins or scaly anteaters), Carnivora (carnivores), Perissodactyla (horses, tapirs, rhinoceroses and other odd-toed ungulates) and Artiodactyla [pigs, cattle, deer and other even-toed ungulates; dolphins and whales (O'Leary et al. 2013)].

Since Dobson's (1875) basal division of Chiroptera into the suborders Megachiroptera (also referred to as 'megabats', Old World fruit bats or flying-foxes) and Microchiroptera ('microbats'), these two groups have been widely accepted. In this arrangement, Megachiroptera comprised only Pteropodidae and Microchiroptera included all other families (Koopman 1994). This classification was widely used for more than a century, but it is no longer accepted by bat systematists. Currently, Yinpterochiroptera (Rhinolophoidea + Pteropodidae) and Yangochiroptera (all other families) are the two most basal lineages recognised within the Chiroptera (Springer et al. 2001, Van Den Busshe & Hoofer 2004). A more in-depth overview of the historical classification of Chiroptera is available in the Supplementary data. Other information about echolocation and ecosystem services provided by bats are available in the Supplementary data as well.

## Important disease outbreaks associated with bats

Following are some examples of viruses of humans and livestock associated with bats and which have been important in many ways, including bringing bats to the attention of the scientific and medical communities. Studies of these diseases, their causative agents, the peculiar biology of bats, the relationships of bat viruses to other viruses, the evolution of these viruses, the causes of disease outbreak initiation (epidemiology), the prevention of diseases and the expanded studies of bats for one reason or another likely will provide more information about the bats themselves and, in the long run, help us protect bat populations from further decline.

*Summary of viruses from bats -* More than 200 viruses have been isolated from or detected in bats. Represented are bats of both suborders, 11 families and 37 genera (Table II, Supplementary Table II). The viruses are representatives of 27 virus families, which is a remarkable diversity (Calisher 2015). This suggests that viruses detected in bats are unlikely to be there by chance, simply a series of oddities. Indeed, when concerted efforts have been made to search for viruses of specific families, such as coronaviruses (CoVs) and herpesviruses, a large number of these viruses were detected and a substantial proportion of them have been shown to be previously unrecognised viruses or viral subtypes. Because some investigators search only for particular viruses or for viruses of particular families it is certain that many viruses must have been overlooked, so that the lists in the Table II and Supplementary Table II surely underrepresent the actual situation *in nature*.

**TABLE II:** Bat hosts from which the virus or its sequence was first identifieda

Family Genus	Virus	Source^b, c^
Adenoviridae		
Mastadenovirus	Ryukyu	Ryukyu flying-fox (*Pteropus dasymallus yayeyamae*)
	Bat adenovirus 2	Common pipistrelle (*Pipistrellus pipistrellus*)
	BtAdV 4	Leschenault’s rousette (*Rousettus leschenaultii*)
Arenaviridae		
Arenavirus	Tacaribe	Jamaican fruit-eating bat (*Artibeus jamaicensis*)
Astroviridae		
Mamastrovirus	Many	Numerous genera and species
Bornaviridae		
Unnamed genus	Bat bornavirus b1	Common pipistrelle (*P. pipistrellus*)
Bunyaviridae		
Orthobunyavirus	Catu	Thomas’s mastiff bat (*Molossus currentium*)
	Guama	Unidentified bat
	Nepuyo	Jamaican fruit-eating bat (*A. jamaicensis*)
	Mojui dos Campos	Unidentified bat
	Kaeng khoi	Wrinkled-lipped free-tailed bat (*Chaerephon plicatus*)
Hantavirus	Araraquara	Tailed tailless bat (*Anoura caudifer*)
		Hairy-legged vampire bat (*Diphylla ecaudata*)
	Hantaan	Common serotine (*Eptesicus serotinus*)
	Huangpi	Japanese pipistrelle** (*Pipistrellus abramus*)
	Longquan	Horseshoe bats (*Rhinolophus* *affinis*,** *Rhinolophus sinicus*,** *Rhinolophus monoceros*)
	Magboi	Hairy slit-faced bat (*Nycteris hispida*)
	Mouyassué	Banana pipistrelle (*Neoromicia nanus*)
	Xuan son	Pomona leaf-nosed bat (*Hipposideros pomona*)
Phlebovirus	Rift Valley fever	Peters’s lesser epauletted fruit bat (*Micropteropus pusillus*)
	Toscana	Kuhl’s pipistrelle (*Pipistrellus kuhlii*)
	Malsoor	Leschenault’s rousette (*R. leschenaultii*)
Nairovirus	Ahun	Whiskered myotis (*Myotis mystacinus*)
	Gossas	Free-tailed bat (*Tadarida *sp.)
Nairovirus	Keterah	Lesser Asiatic yellow house bat (*Scotophilus kuhlii*)
	Issyk-kul	Common noctule (*Nyctalus noctula*)
	Yogue	Egyptian rousette (*Rousettus aegyptiacus*)
	Kasokero	Egyptian rousette (*R. aegyptiacus*)
Caliciviridae		
Sapovirus	Bat sapovirus	Pomona leaf-nosed bat (*H. pomona*)
Circoviridae		
Circovirus	Many	Bats of different species
Cyclovirus	Cyclovirus *T. brasiliensis*	Mexican free-tailed bat (*Tadarida brasiliensis mexicana*)
Coronaviridae		
Alphacoronavirus	Human coronavirus	Sundevall’s leaf-nosed bat (*Hipposideros caffer*)
		Small long-fingered bat (*Miniopterus pusillus*)
	Novel	New Zealand lesser short-tailed bat (*Mystacina tuberculata*)
Betacoronavirus	Severe acute respiratory syndrome coronavirus(SARS)	Chinese rufous horseshoe bat (*R. sinicus*)
	Middle East respiratory syndrome coronavirus	Egyptian tomb bat (*Taphozous perforatus*)
Dicistroviridae		
	Paris dicistrovirus	Common pipistrelle (*P. pipistrellus*)
Filoviridae		
Marburgvirus	Marburg	Egyptian rousette (*R. aegyptiacus*)
Ebolavirus	Zaire	Hammer-headed fruit bat (*Hypsignathus monstrosus*)
Cuevavirus	Lloviu	Franquet’s epauletted fruit bat (*Epomops franqueti*)
		Little collared fruit bat (*Myonycteris torquata*)
		Schreibers’s long-fingered bat (*Miniopterus schreibersii*)
Flaviviridae		
Flavivirus	Bukalasa bat	Little free-tailed bat (*Chaerephon pumilus*)
	Carey island	Lesser short-nosed fruit bat (*Cynopterus brachyotis*)
	Dakar bat	Yellow bat (*Scotophilus* sp.)
	Entebbe bat	Little free-tailed bat (*C. pumilus*)
	Japanese encephalitis	Leschenault’s rousette (*R. leschenaultii*)
	Jugra	Lesser short-nosed fruit bat (*C. brachyotis*)
Flavivirus	Kyasanur Forest disease	Rufous horseshoe bat (*Rhinolophus rouxi*)
	Montana myotis leucoenc.	Little brown myotis (*Myotis lucifugus*)
	Phnom-Penh bat	Lesser short-nosed fruit bat (*C. brachyotis*)
	Rio bravo	Mexican free-tailed bat (*T. brasiliensis mexicana*)
	Saboya	Gambian slit-faced bat (*Nycteris gambiensis*)
	St. Louis encephalitis	Mexican free-tailed bat (*T. brasiliensis mexicana*)
	Sokuluk	Common pipistrelle (*P. pipistrellus*)
	Tamana bat	Common mustached bat (*Pteronotus parnellii*)
	Usutu	Common pipistrelle (*P. pipistrellus*)
	West Nile	Big brown bat (*Eptesicus fuscus*)
	Yellow fever	Little epauletted fruit bat (*Epomophorus labiatus*)
	Yokose	Schreibers’s long-fingered bat (Miniopterus *schreibersii fuliginosus*)
Hepacivirus	Hepatitis C (clade A)	Striped leaf-nosed bat (*Hipposideros vittatus*)
	Hepatitis C (clade C)	Large-eared giant mastiff bat (*Otomops martiensseni*)
	Hepatitis C (clade D)	Striped leaf-nosed bat (*H. vittatus*)
Pegivirus	Gbv-d	Indian flying-fox (*Pteropus giganteus*)
	Pegivirus clades G, H, K	Numerous
Pestivirus	Ra pestivirus 1	Intermediate horseshoe bat (*R. affinis*)
Hepadnaviridae		
Orthohepadnavirus	Unnamed	Schreibers’s long-fingered bat (*M. schreibersii fuliginosus*)
	Hepatitis B	Common tent-making bat (*Uroderma bilobatum*)
	Hepatitis B	Noack’s leaf-nosed bat (*Hipposideros *cf.* ruber*)
	Hepatitis B	Halcyon horseshoe bat (*Rhinolophus alcyone*)
	Kolente	Roundleaf bat (Hipposideros sp.)
Hepeviridae		
Unnamed genus	Hepatitis E virus-like	Bechstein’s myotis (*Myotis bechsteinii*)
Herpesviridae		
(Alpha-herpesvirinae) Simplexvirus	Parixa	Thomas’s nectar bat (*Lonchophylla thomasi*)
(Beta-herpesvirinae) unnamed genus	Agua preta	Gray short-tailed bat (*Carollia subrufa*)
Cytomegalovirus	‘A cytomegalovirus’	Little brown myotis (*M. lucifugus*)
(Gammaherpesvirinae) Percavirus, Rhadinovirus, Macavirus	Many	Bats of different species
Nodaviridae		
Nodavirus	Sers nodavirus	Common serotine (*E. serotinus*)
Orthomyxoviridae		
Influenza virus A	Influenza virus A (H17N10)	Little yellow-shouldered bat (*Sturnira lilium*)
	Influenza virus A	Spix’s artibeus (*Artibeus planirostris*)
Papillomaviridae		
Omegapapillomavirus	MRPV-1	Rickett’s big-footed bat (*Myotis ricketti*)
New genus?	MSPV-1	Schreibers’s long-fingered bat (*M. schreibersii*)
Paramyxoviridae		
Morbillivirus	Canine distemper-like	Common vampire bat (*Desmodus rotundus*)
Henipavirus	Hendra	Gray-headed flying-fox (*Pteropus poliocephalus*)
	Nipah	Variable flying-fox (*Pteropus hypomelanus*)
	Cedar	Black flying-fox (*Pteropus alecto*)
Rubulavirus	Achimota virus 1	African straw-coloured fruit bat (*Eidolon helvum*)
	Mapuera	Little yellow-shouldered bat (*S. lilium*)
	Menangle	Black flying-fox (*P. alecto*)
	Mumps	Epauletted fruit bat (*Epomophorus* sp.)
	Sosuga	Egyptian rousette (*R. aegyptiacus*)
	Tioman	Variable flying-fox (*P. hypomelanus*)
	Tuhokovirus 1	Leschenault’s rousette (*R. leschenaultii*)
	Tuhokovirus 2	Leschenault’s rousette (*R. leschenaultii*)
	Tuhokovirus 3	Leschenault’s rousette (*R. leschenaultii*)
Pneumovirus	Unnamed	African straw-coloured fruit bat (*E. helvum*)
Unassigned	‘A paramyxovirus’	Leschenault’s rousette (*R. leschenaultii*)
Parvoviridae		
Unnamed PARV4-like	Eh-BtPV-1	African straw-coloured fruit bat (*E. helvum*)
Dependovirus	BtAAV-YNM	Rickett’s big-footed bat (*M. ricketti*)
Bocavirus	MmBoV-1	Mouse-eared myotis (*Myotis myotis*)
Undetermined	Aj-BtPV-1	Jamaican fruit-eating bat (*A. jamaicensis*)
Picobirnaviridae		
Picobirnavirus	Unnamed	Common pipistrelle (*P. pipistrellus*)
Picornaviridae		
Kobuvirus	*E. helvum* kobuvirus	African straw-coloured fruit bat (*E. helvum*)
Unclassified	C16A	Multiple bat sources
	Ms picornavirus 1	Schreibers’s long-fingered bat (*M. schreibersii*)
	Ia io picornavirus 1	Great evening bat (*Ia io*)
	Ra picornavirus 1	Intermediate horseshoe bat (*R. affinis*)
	Juruaca	Undetermined bat
Polyomaviridae		
Undetermined	‘A polyomavirus’	Little brown myotis (*M. lucifugus*)
Poxviridae		
Chordopoxvirinae (Molluscipoxvirus)	Molluscum contagiosum-like	African straw-coloured fruit bat (*E. helvum*)
		
Chiropoxvirinae	Eptesipox	Big brown bat (*E. fuscus*)
Reoviridae		
Orbivirus	Fomede	Dwarf slit-faced bat (*Nycteris nana*)
	Ife	African straw-coloured fruit bat (*E. helvum*)
Orthoreovirus	Japanaut	Southern blossom bat (*Syconycteris australis crassa*)
	Broome	Little red flying-fox (*Pteropus scapulatus*)
	Nelson bay	Gray-headed flying-fox (*P. poliocephalus*)
	Pulau	Variable flying-fox (*P. hypomelanus*)
	Xi river	Leschenault’s rousette (*R. leschenaultii*)
Rotavirus	Bat/KE4852/07	African straw-coloured fruit bat (*E. helvum*)
	Maule	Whiskered myotis (*M. mystacinus*)
	RVA/Bat-tc/MYAS33	Stoliczka’s Asian trident bat (*Aselliscus stoliczkanus*)
Retroviridae		
Betaretrovirus	Endogenous	Bats of different species (from genome databases)
Spumavirus	RaFV-1	Intermediate horseshoe bat (*R. affinis*)
Gammaretrovirus	Sers gammaretrovirus	Common serotine (*E. serotinus*)
Rhabdoviridae		
Lyssavirus	Rabies	Common vampire bat (*D. rotundus*)
Lyssavirus	Lagos bat	African straw-coloured fruit bat (*E. helvum*)
	Duvenhage	Schreibers’s long-fingered bat (*M. schreibersii*)
	European bat lyssavirus 1	Common serotine (*E. serotinus*)
	European bat lyssavirus 2	Daubenton’s bat (*Myotis daubentonii*)
	Aravan	Lesser mouse-eared bat (*Myotis blythii*)
	Australian bat lyssavirus	Black flying-fox (*P. alecto*)
	Khujand	Whiskered myotis (*M. mystacinus*)
	Irkut	Greater tube-nosed bat (*Murina leucogaster*)
	West Caucasian bat	Schreibers’s long-fingered bat (*M. schreibersii*)
	Bokeloh virus	Natterer’s myotis (*Myotis nattereri*)
	Shimoni bat	Commerson’s leaf-nosed bat (*Hipposideros commersoni*)
	Lleida bat lyssavirus	Schreibers’s long-fingered bat (*M. schreibersii*)
Vesiculovirus	American bat vesiculovirus	Big brown bat (*E. fuscus*)
Unassigned	Fikirini	Striped leaf-nosed bat (*H. vittatus*)
	Kern canyon	Yuma myotis (*Myotis yumanensis*)
	Mount Elgon bat	Eloquent horseshoe bat (*Rhinolophus eloquens*)
	Oita 296	Little Japanese horseshoe bat (*Rhinolophus cornutus*)
Togaviridae		
Alphavirus	Chikungunya	Unidentified bat
	Sindbis	Pool of roundleaf bat (*Hipposideros* sp.) and horseshoe bat (*Rhinolophus* sp.)
	Cabassou (VEE V)	Unidentified bat
	Venezuelan equine encephalitis (VEE) IA-B	Common vampire bat (*D. rotundus*)
	VEE (IE)	Common tent-making bat (*U. bilobatum*)
	VEE (IF)	Seba’s short-tailed bat (*Carollia perspicillata*)
Totiviridae		
Totivirus	Tianjin totivirus	Faeces from unidentified bats

*a*: adapted with permission from Calisher (2015);

*b*: many of the viruses listed in this Table were first isolated from sources other than bats. The bat hosts listed here are those from which these viruses were first obtained or otherwise detected. Note that certain of the viral nucleic acid sequences detected have been identified to virus family or to genus, but not to species, thus this is a provisional list;

*c*: vernacular names of bats follow primarily Wilson and Reeder (2005). Some of these viruses or sequences have now been detected in bats of other species.

*Rabies -* No review concerning bats and viruses can be complete without at least a brief summary of this first virus recognised in bats and the classical account of the disease it causes. Numerous books and scientific publications are in print or otherwise available which provide details regarding Louis Pasteur's efforts to devise a vaccine against the disease called rabies (L. *rabies*, rage, madness). Classical rabies virus (street rabies) is the prototype virus of a complex of viruses that have been detected in bats, dogs and other mammals. This negative sense, single-stranded RNA virus has been classified in the virus order Mononegavirales, family Rhabdoviridae (Gr. *rhabdos*, rod) and genus Lyssavirus (Gr. *lyssa*, rage, fury, canine madness).

Rabies may be the oldest human infectious disease known. Its origin has been associated with wolves [*Canis lupus* (Carnivora, Canidae)], now domesticated as dogs (*C. lupus familiaris*). Even as far back as 2300 BC dog owners in the Babylonian city of Eshnunna were fined for deaths caused by their dogs having bitten people. In 500 BC, the Greek philosopher Democritus described a case of canine rabies. In 400 BC, Aristotle wrote 'dogs suffer from the madness. This causes them to become very irritable and all animals they bite become diseased', In the first century, Roman writer Cardanus described the saliva from a rabid dog as a 'virus' (L. *virus*, poison). In 1804, a German scientist, Georg Gottfried Zinke, demonstrated that rabies could be passed through saliva from rabid dogs.

Nonetheless, the geographic distribution of all haematophagous bats (vampire bats), either extant or fossil, are restricted to the New World tropics (Koopman 1994). For whatever reasons, whether brought to the New World or because Trinidad and Tobago and Haiti were foci of rabies and inferred associations of rabies and bats, the mythology and superstitions encompassing bats served as an impediment to research on bats and on infectious diseases. To some extent, though considerably lessening, this remains true.

It was Antonio Carini (1911) - an Italian physician, bacteriologist and professor and director of the Pasteur Institute of São Paulo - who presented findings that rabies of herbivores could be transmitted by bats. However, the first isolation of rabies virus was from a common vampire bat [*Desmodus rotundus* (Phyllostomidae)] in 1931 (Baer 1991). The same conclusion drawn by Carini was made by Lima (1934), also in Brazil, and Pawan (1936), in Trinidad; the latter also made the connection between fruit-eating bats and paralytic rabies (Pawan 1948). These studies truly turned the direction of rabies, virus and bat research forward, but it was not until relatively recently that other viruses and bats themselves came into prominence, as mentioned below.

In 1903, Adelchi Negri, an Italian physician, reported his observations of what came to be called 'Negri bodies', eosinophilic inclusions found in the cytoplasm of nerve cells containing rabies virus (Negri 1903). Remarkably, it was not until 1953 that the first American case of rabies in a bat was reported from Pennsylvania (Witte 1954). For many years thereafter, diagnostic techniques began to be improved, epidemiologic investigations expanded, specific monoclonal antibodies produced and applied and investigations of rabies virus put on a molecular basis, but the primary advance in rabies diagnosis was the development of an immunofluorescence test in the 1950s, used to detect rabies virus antigens (Goldwasser & Kissling 1958).

*Rabies virus-related viruses -* Other rhabdoviruses have been isolated from bats (Calisher & Ellison 2012). Aravan virus has been isolated from a lesser mouse-eared bat [*Myotis blythii* (Vespertilionidae)] in Kyrghyzstan, Central Asia, Australian bat lyssavirus, from black (*Pteropus alecto*), gray-headed (*Pteropus poliocephalus*), little red (*Pteropus scapulatus*) and spectacled (*Pteropus conspicillatus*) flying-foxes (Pteropodidae) and yellow-bellied pouched bats [*Saccolaimus flaviventris* (Emballonuridae)], in Australia, Bokeloh virus from a Natterer's myotis [*Myotis nattereri* (Vespertilionidae)] in Germany, Duvenhage virus, from Schreibers's long-fingered bat [*Miniopterus schreibersii* (Miniopteridae)] and Egyptian slit-faced bat [*Nycteris thebaica* (Nycteridae)] in South Africa, European bat lyssavirus 1, from common serotines [*Eptesicus serotinus* (Vespertilionidae)], European bat lyssavirus 2, from Daubenton's bats [*Myotis daubentonii *(Vespertilionidae)], Irkut virus, from greater tube-nosed bats [*Murina leucogaster* (Vespertilionidae)] in Russia and China, Khujand virus, from a whiskered myotis [*Myotis mystacinus* (Vespertilionidae)] in Tajikistan, Lagos bat virus, first isolated from a African straw-coloured fruit bat [*Eidolon helvum* (Pteropodidae)] in Nigeria, Lleida bat lyssavirus, from Schreibers's long-fingered bat in Spain, Shimoni bat virus, from a Commerson's leaf-nosed bat [*Hipposideros commersoni* (Hipposideridae)] in Kenya and West Caucasian bat virus, from a Schreibers's long-fingered bat in Russia. Mokola virus, although not isolated from bats, also is a member of the rabies-virus related virus group and has been isolated from shrews [*Crocidura* spp (Soricomorpha, Soricidae)] and domestic cats in Nigeria. Since the initial detections of these viruses, with the exception of Mokola virus, most have been detected in bats of species other than those mentioned above. Among those mentioned above, only pteropodids feed on plants, whereas all others feed on animals, including insects, other invertebrates or vertebrates. 

Severe rabies-like disease in humans has led to additional studies of flying-foxes, resulting in a greater understanding of the epidemiology and geographic distribution of Australian bat lyssavirus. Based on this evidence, Fraser et al. (1996) suggested that bats may play a more important role in the circulation of virus diseases than had been previously realised.

*Hendra, Nipah and other paramyxovirus diseases -* Hendra and Nipah viruses are both highly pathogenic zoonotic paramyxoviruses (Mononegavirales, Paramy- xoviridae) that have been detected in pteropodid bats within the last decades (Marsh et al. 2012). A horse died of undiagnosed cause in 1994 in Queensland, Australia. Eight to 11 days later depression, anorexia, fever, dyspnoea, ataxia, tachycardia, tachypnoea and nasal discharge was reported in 17 other horses from the same area; 14 of them died or were euthanised. Five and six days, respectively, after the death of the index horse, a stable hand and a horse trainer, both of whom had had close contact with the sick horse's mucous secretions, were diagnosed with influenza-like illnesses. The stable-hand recovered, but the trainer developed pneumonitis, respiratory failure, renal failure and arterial thrombosis and succumbed from cardiac arrest seven days after admission to hospital. A paramyxovirus cultured from his kidney was shown to be identical to viruses isolated from the lungs of five affected horses. The two affected humans and the horses had antibody to the virus and the disease was reproduced in healthy horses following challenge with spleen-lung homogenates from infected horses (Selvey et al. 1995). Scattered other cases caused by this virus were identified, but evidence for its otherwise occurrence were not obtained by testing vertebrates and arthropods in the associated areas until flying-foxes were tested and the virus isolated from blood, foetal tissues, uterine fluids, urine, faeces and saliva (Halpin et al. 2000).

The etiologic agent eventually was named Hendra virus after the Queensland location where the first cluster of cases occurred. More than one-fifth of the flying-foxes in eastern Australia were shown to have neutralising antibody to Hendra virus, as did bats of multiple species of flying-foxes in New Guinea. In 1996 Hendra virus was isolated from a flying-fox (Halpin et al. 2000). Epidemiologic evaluations suggested that horses become infected with Hendra virus *via* direct or indirect contact with infected flying-foxes and humans become infected with this virus *via* direct contact with infected horses. Disease control has been made by preventing contact between flying-foxes and horses. 

A second paramyxovirus detected in flying-foxes is Menangle virus (genus Rubulavirus), responsible for a 1997 zoonotic disease affecting pigs and humans in New South Wales, Australia. Antibodies capable of neutralising Menangle virus were detected in flying-foxes, providing provisional evidence of a bat origin for this virus; the virus later was isolated from black flying-foxes (Barr et al. 2012). Samples of Indonesian bats have revealed the presence of henipavirus and rubulavirus RNAs.

In 1998 yet another paramyxovirus, this one named Nipah virus, was recognised as the etiologic agent of a fatal disease of humans and pigs in Malaysia and Singapore (Chua et al. 2000). By June of the following year more than 100 fatalities among 250 human encephalitis cases were diagnosed in Malaysia and another 11 cases, including one fatality, were diagnosed in Singapore. Initially misdiagnosed as an epizootic of Japanese encephalitis, precious time was lost in controlling this epizoodemic. Control efforts eventually included culling of all pigs on affected farms, an extremely costly measure resulting in the near collapse of the billion-dollar pig-farming industry, heightened animosity between communities and elevated administrative costs in Malaysia.

Nipah virus was shown to be closely related to Hendra virus of Australia (Chua et al. 2000) and, because of their large genomes, their limited homologies with other paramyxoviruses and other unique characteristics, these two viruses were placed in a separate genus (Henipavirus) of the family Paramyxoviridae. Because of the similarity of Nipah and Hendra viruses, flying-foxes were suspected as being somehow involved in the epidemiology of Nipah virus. Neutralising antibodies to Nipah virus were detected in pteropodid bats of five species in Malaysia, suggesting widespread infection in bats there. Soon thereafter the virus was detected in urine of variable flying-foxes (*Pteropus hypomelanus*) and in fruits partially eaten by them, confirming these bats as natural hosts of the virus (Chua et al. 2002). Taken together, the epidemiologic portrait was that climatic and human-driven ecologic changes and locations of pig farms in orchards, which are home to fruit bats, provided settings in which Nipah virus can switch species, from fruit bats to pigs to humans. Nipah virus also has been associated with Lyle's flying-fox [*Pteropus lylei* (Pteropodidae)] in Cambodia (Reynes et al. 2005) and pteropodids and hipposiderids in Thailand (Wacharapluesadee et al. 2005).

In early 2001, an outbreak of febrile illness associated with altered sensorium was observed in Siliguri, West Bengal, India; laboratory investigations did not immediately identify an infectious agent. Nipah virus infection had not been previously detected in India, but because Siliguri is near the border with Bangladesh, where outbreaks of Nipah virus infection had recently been reported, samples obtained during the Siliguri outbreak were retrospectively analysed for evidence of Nipah virus infection. Nipah virus-specific IgM and IgG antibodies were detected in nine of 18 patients. Reverse transcriptase-polymerase chain reaction assays detected Nipah virus RNA in urine samples from five patients. Sequence analysis confirmed that the Nipah virus from humans in Siliguri was more closely related to Nipah virus isolates from Bangladesh than to Nipah virus isolates from Malaysia (Chadha et al. 2006).

In contrast to transmission of Nipah virus from bats elsewhere, transmission in Bangladesh was found to be *via* drinking the sap of date palms [*Phoenix dactylifera* (Arecales, Arecaceae)] and *via* person-to-person route. Nipah virus RNAs detected in Bangladesh are variable in their sequences, suggesting multiple introductions *via* Indian flying-foxes *Pteropus giganteus *(Pteropodiae), which migrate long distances and are found in the Maldives, India, Bangladesh, China, Nepal, Pakistan and Sri Lanka.

RNA of Cedar virus, another henipavirus, was detected in urine of pteropodid bats in Australia in 2009, but little is yet known about this virus (Marsh et al. 2012). Challenge studies with Cedar virus in domestic ferrets [*Mustela putorius furo* (Carnivora, Mustelidae)] and domesticated guinea pigs [*Cavia porcellus* (Rodentia, Caviidae)], both susceptible to infection and disease with known henipaviruses, confirmed virus replication and production of neutralising antibodies, but no clinical disease. Also, the major genetic difference between Cedar virus and Hendra and Nipah viruses lies within the coding strategy of the P gene, known to play an important role in evading the host innate immune system. Preliminary studies indicated that Cedar virus infection of human cells induces a more robust interferon-β response than does Hendra virus. Cedar virus is one that might be studied to develop a human and livestock vaccine.

Intriguing evidence for infection with a henipavirus in African bats was presented by Hayman et al. (2008) who reported finding antibody to henipaviruses in African straw-coloured fruit bats from Ghana. As a follow-up, Drexler et al. (2009) detected henipaviral RNA in an African straw-coloured fruit bat. Clearly, information regarding the geographic distribution and medical and veterinary importance of the henipaviruses and their relationship with bats is not nearly complete.

*Severe acute respiratory syndrome (SARS) -* The hundreds of human case notifications of SARS in Guangdong Province, People's Republic of China in late 2002, then elsewhere in the world, moved bat virus recognition from unanticipated and occasional to well-planned and more methodical. Thereafter the World Health Organization (WHO) put the entire world on alert. SARS cases soon were diagnosed in patients not only in Vietnam, but also in Hong Kong and Canada, and cases were diagnosed in health care workers and household members who had cared for patients with the disease. Many of the cases were traced back through chains of transmission to a health care worker from Guangdong Province who had visited Hong Kong, where he was hospitalised with pneumonia and died. By late April 2003, more than 4,000 SARS cases and 250 SARS-related deaths were reported to the WHO from more than 25 countries. Most of these cases occurred after exposure to SARS patients in health care or household settings.

The WHO coordinated a massive international collaborative effort that included clinical, epidemiologic and laboratory investigations and simultaneously initiated efforts to control the spread of the disease. Attempts to identify the causative agent of the outbreak were successful during 2003, when laboratories in the United States of America (USA), Canada, Germany and Hong Kong isolated a novel CoV (SARS CoV) from SARS patients. Classified as a virus in the order Nidovirales, family Coronaviridae and genus CoV, unlike other human CoVs, this one can be isolated in Vero cells. SARS CoV RNA has frequently been detected in respiratory secretions and convalescent-phase serum specimens from SARS patients contain antibodies that react with SARS CoV, altogether providing evidence that it was a newly recognised virus and associated with the disease. The source of the virus *in nature *had not been determined at that time, but knowing it was a CoV made the search easier.

CoVs comprise a diverse group of large, enveloped, positive-stranded RNA viruses that cause respiratory and enteric diseases in humans and other animals. Their genomes are the largest (about 30,000 nucleotides) of any RNA virus known. A great deal is known about CoVs, nicely summarised by Ksiazek et al. (2003) and Rota et al. (2003) in their papers describing molecular and other characteristics and properties of SARS CoV and comparing its genome to the genomes of other CoVs.

Many possible natural history scenarios - among others, human infections originating with masked palm civets [*Paguma larvata* (Carnivora, Viverridae)] and raccoon dogs [*Nyctereutes procyonoides* (Carnivora, Canidae)] in live markets of wild animals in mainland China - were proposed, but were more confusing than helpful to our understanding of the origin and spread of the virus. Poon et al. (2005), searching for the SARS CoV in Hong Kong bats, came close to succeeding, being the first to detect a CoV (group 1, i.e., alphacoronaviurs) in bats; a retrospective study of samples collected for other purposes demonstrated the presence of an alphacoronavirus RNA sequence in an Australian bat captured in 1996 (LL Poon, unpublished observations). Then Lau et al. (2005) and Li et al. (2005) reported detections of (group 2, i.e., betacoronavirus) SARS CoV-like viruses in bats, thus providing evidence that bats are a natural source of at least some of the numerous alphacoronavirus and betacoronavirus found world-wide (Osborne et al. 2011). By now, partial descriptions of many hitherto unrecognised coronaviral sequences have been published in the scientific literature. Without more biological and epidemiological information it is difficult to determine whether these represent newly recognised viruses, are closely or distantly related strains or are more items to add to lists; these are not tabulated in Table II. Obviously, evidence of the presence of many important viruses in bats has served to invigorate studies of the biology of bats themselves as well as the discovery of disease-associated viruses.

*Middle East respiratory syndrome (MERS) -* MERS is caused by a CoV called MERS CoV. Cases of this disease were first reported from Saudi Arabia by ProMED and formally published soon after (Bermingham et al. 2012). MERS affects the respiratory system and most MERS patients develop severe acute respiratory illness with fever, cough and shortness of breath. The case-fatality rate of MERS is about 45%. Through retrospective investigations, health officials later showed that the first known cases of MERS occurred in Jordan in 2012. Thus far, all cases of MERS have been linked to countries in and near the Arabian Peninsula. This virus has spread from ill people to others through close contact, such as caring for or living with an infected person. As of 23 January 2015, there have been 956 laboratory confirmed cases of MERS CoV infection, including 351 deaths, a large proportion of whom had pre-existing co-morbidities. Because of its similarity to the SARS CoV, it had been anticipated that bats were somehow involved in transmission of the MERS CoV and Memish et al. (2013) detected a partial RNA sequence of a betacoronavirus with 100% identity to virus from the human index case-patient. This nucleotide sequence was obtained from a faecal pellet from an Egyptian tomb bat [*Taphozous perforatus* (Emballonuridae) (Memish et al. 2013)] in Saudi Arabia, and a close relative of this virus was detected in a Zulu serotine bat [*Neoromicia *cf.* zuluensis* (Vespertilionidae)] by Ithete et al. (2013) in South Africa. However, recent studies have suggested that one-humped camels [*Camelus dromedarius* (Artiodactyla, Camelidae)] may be a primary source of this virus *in nature *(Raj et al. 2014) and experimental infections of camels with MERS CoV seem to support this view (Adney et al. 2014). MERS CoV continues to cause disease in the Arabian Peninsula, but is not expected to cause a pandemic.

*Marburg and ebolavirus haemorrhagic fevers -* Marburg and ebolaviruses - (Mononegavirales, Filoviridae), genera Marburgvirus and Ebolavirus, respectively, the 'filoviruses' - were discovered because they cause severe, often fatal, haemorrhagic diseases in humans and other primates.

*Marburgvirus disease -* In late summer of 1967, an haemorrhagic fever outbreak was observed in laboratory workers in Serbia, at the time part of Yugoslavia and in Germany. It was soon shown that the disease was transmitted from green monkeys [*Chlorocebus sabaeus* (Primates, Cercopithecidae)] consigned from Uganda to Europe, but the origin of the disease was unknown, other than that it was caused by a hitherto unrecognised virus, named Marburg virus for the city in Germany where the disease was first recognised. Another infection with this virus occurred in a traveller in Africa in 1975 and cases of Marburg haemorrhagic fever have been documented with some frequency in various parts of Africa since then. Although a great deal was learned about this virus from pathologic and laboratory studies, its epidemiology remained undetermined. Nonetheless, bits and pieces of evidence suggested that bats might be associated with Marburg virus. It was not until 1999 that Swanepoel et al. (2007) detected Marburg virus RNA in Egyptian rousettes, eloquent horseshoe bats [*Rhinolophus eloquens* (Rhinolophidae)] and a greater long-fingered bat [*Miniopterus inflatus* (Vespertilionidae)] captured in the Democratic Republic of the Congo and Towner et al. (2009) isolated genetically diverse Marburg viruses from Egyptian rousettes. Marburgviruses are now placed in the genus Marburgvirus, species Lake Victoria marburgvirus.

*Ebolavirus diseases -* In 1976, a series of severe and often fatal haemorrhagic fevers occurred in southern Sudan. Almost immediately after those cases were recognised, a similar disease was observed in humans in Zaire, now known as the Democratic Republic of the Congo, nearly 1,000 km away. A virus, originally termed 'Ebola virus', named after the Ebola River near the epidemic site in Zaire, was isolated from patients in Zaire and partially characterised (Johnson et al. 1977) but, as with Marburg virus, early intensive field studies did not reveal the source of the virus. The virus detected in Zaire eventually was named Zaire ebolavirus and the distinct virus from Sudan was named Sudan ebolavirus.

A third ebolavirus, Reston ebolavirus, was discovered in 1989 when an outbreak of anorexia, nasal discharge, splenomegaly and haemorrhaging was recognised among crab-eating macaques [*Macaca fascicularis* (Primates, Cercopithecidae)] imported from a commercial source in the Philippines to a primate holding site in Reston, Virginia, USA. No human illnesses among animal handlers were recognised (Jahrling et al. 1990). Then, in 1994, clinical investigations into the haemorrhagic illnesses and deaths of common chimpanzees [*Pan troglodytes verus* (Primates, Hominidae)] in the Taï Forest of Côte d'Ivoire resulted in an accidental infection and non-fatal illness of an investigator performing a necropsy on one of these primates. Virus isolation from this patient was successful and the virus named Taï Forest ebolavirus. The cause of deaths of the members of the chimpanzee group was later shown to be infection with this virus. This was the first time that a human ebolavirus infection had been connected to naturally-infected non-human primates in Africa (Le Guenno et al. 1995).

Epidemiologic and virologic investigations of an epidemic of haemorrhagic fever in humans in western Uganda in 2007 revealed the cause as being Bundibugyo ebolavirus, named for the district in Uganda where the epidemic occurred. This virus is more closely related to Taï Forest ebolavirus than it is to other ebolaviruses, but is distinct from them all (Towner et al. 2008).

In sum, since 1967 filoviruses have been the cause of very serious and focal or widespread haemorrhagic fever outbreaks in Africa, as well as in the USA, although the latter experience was limited to imported monkeys. Most of the haemorrhagic fever outbreaks or epidemics have had case-fatality rates of 50-90%. The identified index cases in these situations have been shown to have been in contact with ebolavirus-infected primates or other large vertebrates, either killed or found dead in forested areas. Because bats have been shown to harbour various filoviruses and because they are used as protein sources by human and free-ranging non-human-primates in some parts of Africa, Asia and perhaps elsewhere, eating and other contacts with an ebolavirus may result in virus transmission and epidemic initiation. The validity of such a scenario remains to be proven.

A novel filovirus, provisionally named Lloviu virus (the only virus in the genus Cuevovirus), was detected during the investigation of bat die-offs in Cueva del Lloviu in Spain in 2002. Lloviu virus is genetically distinct from marburgviruses and ebolaviruses and is the first filovirus detected in Europe that was not imported from an endemic area in Africa. Whereas infections of bats with marburgviruses and ebolaviruses do not appear to be associated with disease in the bats, Lloviu virus was detected in a dead Schreibers's long-fingered bat.

Survival of bats from experimental infections led Swanepoel et al. (1996) to list Wahlberg's epauletted fruit bats [*Epomophorus wahlbergi* (Pteropodidae)], little free-tailed bats [*Chaerephon pumilus* (Molossidae)] and Angolan free-tailed bats [*Mops condylurus* (Molossidae)] as potential hosts for Zaire ebolavirus. In addition, from 2001 and 2003, Leroy et al. (2005) collected small vertebrates at sites where non-human primates had died at the border between Gabon and the Republic of the Congo. They detected RNA of an ebolavirus in hammer-headed fruit bats (*Hypsignathus monstrosus*), Franquet's epauletted fruit bats (*Epomops franqueti*) and little collared fruit bats (*Myonycteris torquata*). These RNA sequences were quite similar to those of the ebolavirus isolated from humans during the 1976 outbreak in Zaire. Other investigations led to antibody detections in bats of the same species. Leroy et al. (2005) detected ebolaviral RNA from them and from other pteropodids and molossids as well (Olival & Hayman 2014). This has been the ostensible link for an association of bats with Zaire ebolavirus and conﬁrmed their speculation that ebolaviruses circulate in the forests of Central Africa (Leroy et al. 2005). The West African ongoing outbreak that is ravaging Guinea, Liberia and Sierra Leone, initiated more than one year ago, has sickened more than 20,000 people and killed more than 8,000 of them to date. Although these countries are geographically closer to the Ivory Coast than the Democratic Republic of Congo and South Sudan, Baize et al. (2014) demonstrated that this strain is phylogenetically closer to the Zaire ebolavirus than is the Taï strain. Gire et al. (2014) provided support for this hypothesis, their data suggesting that the West African variant diverged from the Zaire ebolavirus (central African lineage) about 2004.

Based on the work of Leroy et al. (2005), frugivorous bats were tentatively linked to the ongoing outbreak in West Africa (Baize et al. 2014), but field investigations raised a new scenario for the emergence of the virus. According to Saéz et al. (2015), the index case (a 2-year-old boy from the small village of Meliandou, Guinea) may have been infected by playing in a hollow tree used to house a colony of Angolan free-tailed bats - a species widespread in Central and West Africa. The tree was burned and the colony no longer lives in the hollow. During field investigations Saéz et al. (2015) found no infected bats; however, Angolan free-tailed bats are among those that have survived experimental infections (Swanepoel et al. 1996). Thus, the accumulated evidence, while implicating bats in transmission of many viruses, may only be coincidental and remains unproven.

## Bat immunology

In addition to their other well-known uniquenesses, it may be asked whether bats have unique responses to virus infections. That is, why (or how) do bats survive infections with viruses and other agents that are pathogenic for other vertebrates? Or do they? These questions have not been answered satisfactorily and data accumulated thus far have not definitively answered them, but have led to speculation, much of which is fascinating. Serologic studies demonstrated that bats have antibody to many viruses, but it was not determined whether their antibody isotypes were similar to those of other mammals. Nearly 50 years ago, for example, Sulkin et al. (1966) maintained big brown bats [*Eptesicus fuscus *(Vespertilionidae)] experimentally infected with Japanese encephalitis virus at various environmental temperatures and then tested them for both virus and neutralising antibody. Viraemias were demonstrable in most of these bats within two-three days after infection and persisted for one-two weeks. However, not all viraemic bats produced antibody to the virus, a few others had equivocal and then delayed (nearly 2 months) antibody responses and some that had produced antibody soon after being infected no longer had detectable antibody three months later.

We know that bats produce IgM, IgG, IgA and IgE antibodies, but not whether these bat immunoglobulins react as do similar immunologic isotypes of other vertebrates. There are clear differences between bats of different species. For example, only a single IgG subclass has been identified in Seba's short-tailed bats [*Carollia perspicillata* (Phyllostomidae)], whereas little brown myotis [*Myotis lucifugus* (Vespertilionidae)] have five IgG subclasses (Butler et al. 2011). One can conclude from this that bats of different species differ, which is not enlightening taxonomically, but is informative in regard to the danger of making generalisations about bats.

Now that bats have been shown to host hundreds of viruses, some of them important pathogens of humans, interest in and studies of the immunological responses of bats has increased; data accumulated recently have been fascinating and informative (Baker et al. 2013a, Epstein et al. 2013, Zhang et al. 2013). Baker et al. (2013b) have nicely reviewed the literature concerning antiviral immune responses in bats and the reader is encouraged to see that publication.

Dead or sick bats have been the source of viruses of bats (Supplementary Table II), but these have principally been focused on rabies virus because of the importance of that virus and because of the attendant emphasis on studies of its occurrence, geographic distribution and genotype. Finding dead or sick bats with other viruses is not a common occurrence, likely due to the rapid scavenging of dead animals under natural conditions, although meticulous searching may indicate otherwise (Mühldorfer et al. 2011). Such studies may provide information useful to our understanding of sex differences, seasonality and other aspects of the prevalence of viruses in bats. Even the highly pathogenic rabies virus has been detected in apparently healthy bats (Davis et al. 2012). How rabies virus and other viruses persist over time certainly depends on a mechanism for such persistence, but that mechanism is unclear at this time, although it is being slowly revealed (Blackwood et al. 2013).

Nonetheless, it is unusual to find dead or sick bats. That at least some viruses have been detected in apparently healthy bats, which must be netted or otherwise sampled in the field, has led to hypotheses that centre on the possibility that bats somehow tolerate at least certain virus infections and this has encouraged investigators to question whether bats can allow virus infections, remain asymptomatic, yet respond to such infections differently from the ways in which other mammals respond to them.

In an effort to provide a foundation for understanding adaptations by bats that might allow them to at least somewhat peacefully coexist with viruses that infect them, Papenfuss et al. (2012) assembled transcriptome sequences from immune tissues and cells of black flying-foxes. They identified 18,600 genes, of which 650 (3.5%) corresponded to immune genes and about 500 of which were identified, providing information regarding innate and adaptive immunity of these bats. These and other results suggested that bats have many genes consistent with those in available databases, but which may represent bat-specific transcripts. Alternatively, a transcriptome data set of Shaw et al. (2012) representing Jamaican fruit-eating bats [*Artibeus jamaicensis* (Phyllostomidae)] differs somewhat from that of Papenfuss et al. (2012), indicating yet another difference between bats of different species.

Other studies of bat genomes have indicated differences in genes associated with early immune responses, which might allow virus to replicate in the absence of a primary and hearty immunological response. It is further reasonable to assume that this in turn might allow at least low-level virus replication over an extended period. There is some evidence that these genes may be evolving more rapidly in bats than in vertebrates of other taxa, which would suggest that the genes are co-evolving with the bats in response to viral and other infections. An additional note of caution: when studying viruses from bats in order to understand their effects, it is clearly more appropriate to use bats or their cells in culture, rather than other hosts or cells from other hosts. That is, simply because a virus or other pathogen might interfere with or activate a gene product in one host, there is no certainty that it does the same in another host; thus the extrapolatory and speculatory nature of many available publications. A recent paper by Zhang et al. (2012) provides a new insight into the interplay between bat host and infecting virus. The authors isolated a novel β-herpesvirus from a Schreibers's long-fingered bat and, in determining its complete DNA genome sequence, found it to be the first virus genome known to encode major histocompatibility complex (MHC) class II homologues. The authors proposed future functional studies of these MHC class II homologues to determine whether they may play a role in potentially novel virus immune evasion pathways (Zhang et al. 2012). This remarkable finding may change the way we view both bats as hosts and viruses as pathogens.

Humans and most other mammals respond to infections by activating their immune systems at the time they are infected, usually producing type I and type III interferons, which are temporising responses activated prior to humoral antibody responses. The immune systems of bats of at least one species (black fling-fox), on the contrary, appear to be perpetually switched on at a low level, thus allowing them to respond quickly to an infectious agent (Zhou et al. 2014). Alternatively, bats may have a potent innate or inherent immunity, one that is more efficient in restricting viral replication or even restricting innate immunity (Siu et al. 2014).

Hibernation - the state of inactivity in which the heart rate, body temperature and breathing rate are decreased in order to conserve energy - has been put forth as a contributor to viral persistence in bats. Bouma et al. (2010) have summarised the physiological and immunological effects of hibernation as also including depressed metabolism, lower numbers of circulating leukocytes, lower complement levels, decreased response to lipopolysaccharides and lower phagocytotic capacity, cytokine production, lymphocyte proliferation and antibody production. They suggest that hibernation may increase infection risk in bats. Further, it has been proposed that the rapid reestablishment of immune responses in bats emerging from hibernation and infected with the white-nose syndrome fungus, *Pseudogymnoascus destructans* (Ascomycota, Pseudeurotiaceae), may bring about a severe immune reconstitution inflammatory syndrome (Meteyer et al. 2012).

Hibernation also may allow not only virus persistence in the bat, but trans-seasonal persistence as well, allowing virus to amplify and re-emerge when conditions are more amenable to transmission, such as seasonally for arthropod-borne viruses, colony formation and movement to maternity caves.

## Concluding remarks

*Bats and emerging viruses -* More than 200 viruses of 27 families were isolated from or detected in bats of both suborders. A few of these viruses have been responsible for human diseases, including isolated events or disease outbreaks that have resulted in human deaths and bats have been tentatively blamed for some of these episodes (e.g., ebolavirus disease, SARS, MERS). Because bats represent such a large proportion of mammals (about 20%) and are so diversified in their biology, habitats and natural history, it seems reasonable to assume that they have many hundreds more viruses, just as do other diversified groups of life forms. A few hypotheses have been raised to explain so many viruses in bats (O'Shea et al. 2014, Brook & Dobson 2015). However, as to whether these viruses are important or not, whether bats are simply incidental hosts of viruses and whether they serve as competent reservoir hosts of viruses and transmit them to other vertebrates are open questions that must be carefully addressed.

In general, the supposed connections between bats and disease outbreaks caused by zoonotic viruses have been raised more on speculation than on evidence supporting their direct or indirect roles in the epidemiology of those diseases (Fenton et al. 2006). In most cases, the only evidence is the isolation or otherwise detection of the same viruses in bats and humans in areas where the diseases have emerged, but this does not mean that bats are the hosts for the viruses. The identification of the same virus in bats and humans might only be evidence that as mammals they are similar enough to serve as temporary hosts for the virus and the same virus can also be found in other terrestrial vertebrates (e.g., primates, antelopes, birds) and arthropods (Calisher et al. 2006, Melaun et al. 2014). As an example, Ross River virus has been isolated from gray-headed flying-foxes, but these bats did not produce viraemia of sufficient magnitude to be considered competent reservoir hosts for this virus (Ryan et al. 1997). Alternatively, Seymour et al. (1978a) detected specific antibodies of Venezuelan equine encephalitis virus in bats of seven different species from Guatemala. Based on this evidence, Seymour et al. (1978b) experimentally infected bats of five species. Viraemia was detected in most bats, but all were asymptomatic for the infection. They concluded that the phyllostomids Jamaican fruit-eating bats, great fruit-eating bats (*Artibeus lituratus*) and little yellow-shouldered bats (*Sturnira lilium*) had circulating virus levels high enough to infect culicid mosquitoes (Diptera, Culicidae) that may serve as vectors for the virus, whereas great spear-nosed bats [*Phyllostomus hastatus* (Phyllostomidae)] did not develop sufficient viraemia to infect subsequently feeding mosquitoes (Seymour et al. 1978b). Based on these results, we can speculate that there is variability of the immune responses of bats to virus infections, particularly in representatives of closely related species.

Among the scores of viruses and viral sequences identified from bats (Table II), several have been isolated or detected in bat tissues or excreta. However, this does not prove a relationship between the presence of a virus (or its nucleic acid sequence, albeit a nucleotide sequence is not a virus) and the disease the virus might cause. Some of these viruses or viral sequences might have been in food eaten by bats and at least some (or most) are irrelevant with respect to viral disease epidemiology.

Because of the many gaps in our knowledge linking bats and zoonotic viruses, associating bats with these events without any further evidence is a disservice, with negative consequences for bats and humans. For bats, because it puts them on target for 'control' and it disseminates fear among the general public. For humans, because putting efforts to control the wrong reservoir or disease carrier can postpone appropriate mitigation actions that could avoid more deaths or interrupt the spread of the disease and because a potential 'control' of bat populations may deny us their important ecosystem services.

As mammals, bats share many physiological and immunological traits with humans. Additionally, they are in constant contact with humans, with bats of different species roosting in human-made constructions, feeding or hanging on fruit trees or flying around light poles to feed on insects in urban and rural areas. These habits increase their contact with humans, domestic animals, livestock and wildlife, potentially favouring spillovers.

After analysing most of the disease outbreaks that have been tentatively linked to bats, it is obvious that there are at least two transmission routes: from bats directly to humans and from bats to humans through intermediate hosts or vectors. The first one seems to be possible *via* bites during occasional interactions with bats in roosts or bats on the ground (Saéz et al. 2015) and through contact with bat fluids during capturing and preparing them as food (apes, other primates and carnivores can be infected while feeding on bats). However, transmission by contact or ingestion of infected droppings in bat roosts cannot be discounted (Saéz et al. 2015), inasmuch as viruses or viral nucleic acid sequences have been retrieved from urine and faeces of bats (Halpin et al. 2000). The disease caused by the Zaire ebolavirus is a potential example of this route.

Virus transmission from bats to humans through intermediate hosts seems reasonably and logically the link to explain the outbreaks of Nipah and Hendra diseases. However, one aspect of these links has been overlooked. Only animals phylogenetically closer to bats appear as intermediate hosts. Most spillover episodes had horses and pigs linking bats and humans. In other events, camels and carnivores (ferrets, palm civets and raccoon dogs) also figure among the mammals in which viruses apparently circulate. Except for humans, these mammals are from groups that have evolved within the superorder Laurasiatheria. Horses are representatives of the order Perissodactyla, pigs and camels are in the order Artiodactyla and ferrets, palm civets and raccoon dogs are in the order Carnivora; all are members of the superorder Laurasiatheria.

**Fig. 3:**
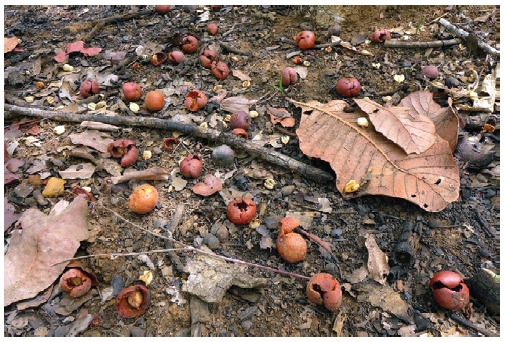
sugar plum (Uapaka kirkiana; Phyllanthaceae) with bat tooth marks on fruits husks, Zambia. Courtesy of Jakob Fahr (Max Planck Institute for Ornithology, Germany), the copyright holder of the image.

Although bats have been distinct from other mammals for more than 52 million years, bats, carnivores perissodactyls and artiodactyls share part of their evolutionary history that is not shared with other mammals and their ancestors may have co-evolved with ancient lineages of certain viruses. This suggests that they may share physiological characteristics that could facilitate circulation of these viruses. Dobson (2006) visited pig farm sites where outbreaks of Nipah virus infections occurred in Malaysia and found partially eaten fruits with bat teeth marks in them. According to Dobson (2006), pigs may have been contaminated by eating fruits that had been partly eaten by bats and subsequently passed the virus to humans. This hypothesis was tentatively rejected by Fenton et al. (2006) based on the argument that pellets rejected by frugivorous bats are composed of indigestible fibres and seeds that have little nutritional value for animals. However, one of us (RM) several times has found on the ground fruits that had been partially eaten by bats (e.g., Fig. 3); there is no reason to believe that other frugivorous or omnivorous mammals, such as primates and carnivores, do not feed on them. Given that Chua et al. (2002) detected Nipah virus in fruits partially eaten by variable flying-foxes, this general scenario seems very reasonable. Using Dobson's (2006) hypothesis as a background, pigs became infected with Nipah virus by eating virus-contaminated fruits and then humans became infected by spillover from pigs, which may have occurred by contact with massive quantities of viruses in the mucosae, faeces or excreta of infected animals. Haematophagous insects may also be virus vectors. Melaun et al. (2014) provided an extensive list of viruses that have been identified in bats, haematophagous insects and humans. With bats spending most of their time stationary and upside down, some of them forming large groups, they are perfect targets for feeding arthropods, particularly mosquitoes. However, it is unlikely that arthropods are important vectors of filoviruses, paramyxoviruses and many other viruses, including rabies virus, as they have not been detected in them even though millions of them have been tested. Mechanical transmission of viruses, however, is always a possibility, but is irrelevant when discussing natural hosts.

The taxonomic and geographic distribution of bats whose viruses have potentially jumped to humans is also noteworthy. Except for rabies, all episodes are in the Old World and pteropodid, hipposiderid and rhinolophid bats - representatives of families in the suborder Yinpterochiroptera - have been mostly involved (Table II, Supplementary Table II). To date, there is no direct evidence that New World bats play important roles in the transmission of zoonotic viruses, other than rabies virus, to humans. This might be caused by a sampling bias because most studies have been concentrated in the Old World tropics; but most studies are concentrated in the Old World because people and livestock are dying there from those diseases. However, it may not be a biased view. Differences in the evolutionary history and biology of New and Old World bats and in human behaviour may provide reasonable explanations. From an evolutionary viewpoint, Old World bats occur in a region where the mammalian fauna was established long ago and those bat lineages may have coevolved with viruses that may have been present in other groups of mammals. In this scenario, bats might have developed strategies to survive virus infections. On the other hand, the assemblage of New World mammals (in particular that from South and Central America) was dramatically reshaped about three million years ago due to the Great American Biotic Interchange (Marshall et al. 1982) and bat and virus lineages may not have had time to coevolve. In addition, all New World bats are in the suborder Yangochiroptera, whereas pteropodids, hipposiderids and rhinolophids are in the suborder Yinpterochiroptera. The basal divergence among these suborders is almost as old as the divergence of bats and other mammals (Springer et al. 2001). Thus, we speculate that bats in these two groups may have evolved distinct immunological characteristics that provide distinct responses to pathogens, with direct implications regarding their roles as hosts. From an ecological viewpoint, some pteropodid bats tend to form large aggregations that may favour virus spread, whereas New World fruit bats usually form smaller aggregations. Also, except for a few native indigenous peoples, in the New World bats are not on the menu, which is different from Africa and Asia where these animals are consumed regularly. Eating them seems not to be a problem because their meat is cooked. The problem appears to be in the contact with their infected blood and other body fluids during handling and preparing their meat. Contact with massive quantities of viruses unquestionably favours spillovers. The above suggestions here are no more than speculations pending formal assessment.

*Future investigations -* We still know comparatively little about the immunological and physiological systems of wild mammals of different orders. With bats being potential reservoirs of a long list of viruses (Table II, Supplementary Table II) and possibly harbouring more zoonotic viruses than mammals of any other group (Luis et al. 2013), including some deadly viruses (Wynne & Wang 2013), it is necessary to understand the mechanisms of immune resistance that allow bats to harbour pathogens, the pathogenetic bases of infectious diseases in bats and the mechanisms underlying disease emergence (Calisher et al. 2006, Dobson 2006, Daszak et al. 2013, Mandl et al. 2015). To address these issues, it is necessary to perform eco-epidemiological field studies and laboratory experiments using bats and bat cell cultures. Bat cell lines derived from tissues of bats of different taxa and strains of laboratory animals need to be established to provide the necessary conditions for in vitro and in vivo experiments.

To develop strains of laboratory animals, several aspects of the biology, natural history and distribution of the potential models must first be considered. To diminish risks of accidental species introductions and minimise effects of potential accidental releases into the native fauna, we suggest selection of autoctone species (those from the local fauna) that have continental distributions as potential models to test enzootic (or endemic) viruses. As an example, Pallas's mastiff bats [*Molossus molossus* (Molossidae)] are potential models for research in South America. They are widely distributed on the continent, adapt well to human constructions and feed on insects (Nowak 1994, Eger 2008), making them easy to maintain in captivity. We suspect that under controlled conditions their reproductive rates may be maximised and, after a few generations, physiologically uniform strains could be obtained.

In addition, fieldwork is necessary to continually searching for new pathogens and to understand the mechanisms underlying the dynamics of zoonotic diseases. It is important to design field studies to understand the role of different biotic and abiotic factors affecting bat populations and pathogen circulation in bats and how these factors may favour spillovers to humans (e.g., habitat disruption, faunal poverty, climate change) (Chua et al. 2002, Parrish et al. 2008, Daszak et al. 2013, Wynne & Wang 2013, Saéz et al. 2015).

Joining expertise from bat biologists and medical investigators, this scenario can quickly move forward to a new one in which the role of bats in the circulation of zoonotic viruses and other pathogenic agents will be at least minimally understood. After understanding the role of bats (or other animals) in the maintenance and circulation of pathogens and the mechanisms underlying the emergence of zoonotic diseases, wildlife biologists and epidemiologists should work together to develop appropriate management plans to control virus circulation and minimise risks of human infection without causing significant biases against specific animal populations. We cannot ignore the potential role of bats in the maintenance, circulation and transmission of pathogens to humans and we cannot ignore the important ecosystem services provided by these animals. Thus, the only possible approach is to develop responsible research to avoid the obstacles and keep safe both human and bat populations. In addition, wildlife biologists and medical investigators should work together to provide expertise to wildlife epidemiologists. This training could be provided by graduate programs in public health.

Finally, can we confidently link bats with emerging viruses? No, or not yet, is the qualified answer based on the evidence available. Only integrative and organised field and laboratory research, using ecological and epidemiological approaches conducted by bat biologists and medical researchers, will provide a useful and satisfactory solution.
